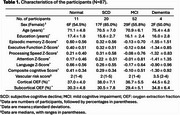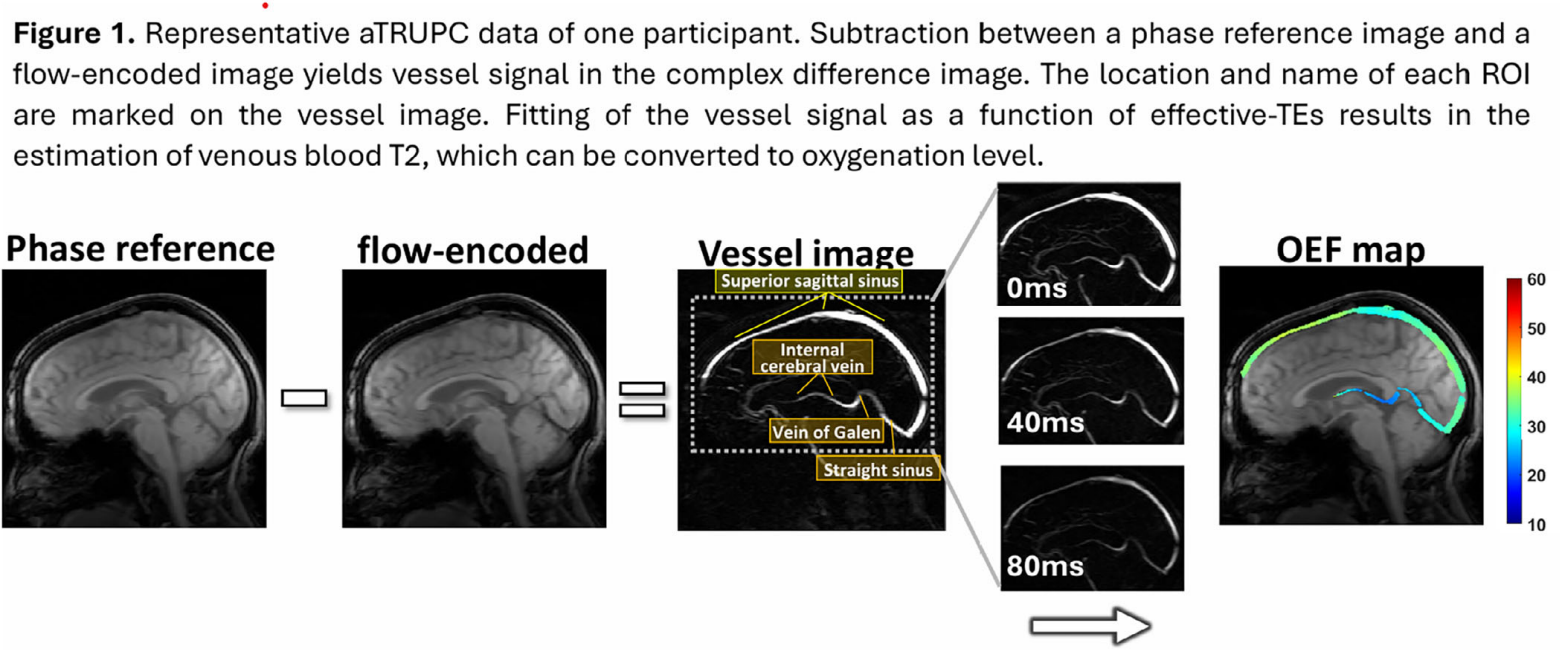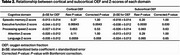# Elevated brain oxygen extraction fraction (OEF) as a potential marker for vascular cognitive impairment and dementia

**DOI:** 10.1002/alz70856_100520

**Published:** 2025-12-24

**Authors:** Jie Song, Wen Shi, Julia Suconic, Kaisha Hazel, George Pottanat, Ebony Jones, Cuimei Xu, Kumiko Oishi, Yifan Gou, Paul B. Rosenberg, Rita Kalyani, Abhay Moghekar, Sevil Yasar, Doris Lin, Marilyn S. S. Albert, Hanzhang Lu, Dengrong Jiang

**Affiliations:** ^1^ Department of Biomedical Engineering, Johns Hopkins University School of Medicine, Baltimore, MD, USA; ^2^ Department of Radiology, Johns Hopkins University School of Medicine, Baltimore, MD, USA; ^3^ Department of Psychiatry and Behavioral Sciences, Johns Hopkins University School of Medicine, Baltimore, MD, USA; ^4^ Division of Endocrinology, Diabetes, and Metabolism, Johns Hopkins University School of Medicine, Baltimore, MD, USA; ^5^ Department of Neurology, Johns Hopkins University School of Medicine, Baltimore, MD, USA; ^6^ Division of Geriatric Medicine and Gerontology, Johns Hopkins University School of Medicine, Baltimore, MD, USA; ^7^ Department of Neurology, The Johns Hopkins University School of Medicine, Baltimore, MD, USA

## Abstract

**Background:**

Vascular cognitive impairment and dementia (VCID) is the second most common cause of cognitive impairment in older adults. However, identifying reliable biomarkers for VCID remains challenging. Cerebral Oxygen‐extraction‐fraction (OEF), a key hemodynamic parameter of brain function, has been found elevated in cerebrovascular disorders such as carotid steno‐occlusive disease and ischemic stroke. In this study, we employed a novel non‐invasive, non‐contrast MRI technique to measure OEF in a VCID‐enriched cohort. We hypothesized that elevated OEF would be associated with worse cognition and vascular risk factors in this cohort.

**Method:**

Eighty‐seven older adults were enrolled from the MarkVCID2 cohort, which is enriched for vascular risk factors and brain white matter lesions and/or microbleeds. Table 1 summarizes the demographic information. Cortical and subcortical OEF values were measured using a technique called accelerated‐T2‐relaxation‐under‐phase‐contrast (aTRUPC) MRI. A comprehensive neuropsychological battery provided five cognitive domain scores: episodic memory, executive function, processing speed, attention, and language. A composite cognitive score was derived by averaging Z‐scores across all domains. A composite vascular‐risk‐score (VRS) was obtained from the number of vascular risk factors present in each patient. Linear regression models were used to analyze the associations between regional OEF, cognitive scores, and VRS.

**Result:**

Figure 1 displays representative MRI. OEF was negatively correlated with the composite cognitive score (cortical: β=‐0.035±0.010, *p* = 0.0012; subcortical: β=‐0.028±0.011, *p* = 0.0093). We next examined the associations between domain‐specific Z‐scores and regional OEF, as summarized in Table 2. After Bonferroni correction, only executive function showed a significant negative correlation with OEF (cortical: β=‐0.044±0.012, *p* = 0.0043; subcortical: β=‐0.038±0.012, *p* = 0.030). Notably, elevated OEF was associated with declines in executive function (characteristic of VCID) but not with declines in episodic memory (characteristic of Alzheimer's disease). These findings suggest that elevated OEF is primarily related to VCID rather than Alzheimer's dementia. Additionally, we observed that OEF was positively associated with VRS (cortical: β=1.451±0.610, *p* = 0.020; subcortical: β=1.335±0.605, *p* = 0.030), further supporting the relationship between elevated OEF and neurovascular pathology.

**Conclusion:**

We showed that elevated cortical and subcortical OEF was associated with poorer cognition (particularly in executive function) among older adults with vascular risks, suggesting that OEF may be a useful biomarker for VCID.